# From Pathogenesis to Therapy in Knee Osteoarthritis: Bench-to-Bedside

**DOI:** 10.3390/ijms22052697

**Published:** 2021-03-07

**Authors:** Elena Rezuş, Alexandra Burlui, Anca Cardoneanu, Luana Andreea Macovei, Bogdan Ionel Tamba, Ciprian Rezuş

**Affiliations:** 1 Department of Rheumatology and Physiotherapy, “Grigore T. Popa” University of Medicine and Pharmacy, 700115 Iaşi, Romania; elena.rezus@umfiasi.ro (E.R.); anca.cardoneanu@umfiasi.ro (A.C.); luana.macovei@umfiasi.ro (L.A.M.); 2 Advanced Center for Research and Development in Experimental Medicine (CEMEX), “Grigore T. Popa” University of Medicine and Pharmacy, 700454 Iaşi, Romania; 3 Department of Internal Medicine, “Grigore T. Popa” University of Medicine and Pharmacy, 700115 Iaşi, Romania; ciprian.rezus@umfiasi.ro

**Keywords:** osteoarthritis, knee joint, disease modifying drugs, cartilage, bone remodeling, inflammation, platelet-rich plasma, mesenchymal stem cells, ozone, hyaluronic acid

## Abstract

Osteoarthritis (OA) is currently the most widespread musculoskeletal condition and primarily affects weight-bearing joints such as the knees and hips. Importantly, knee OA remains a multifactorial whole-joint disease, the appearance and progression of which involves the alteration of articular cartilage as well as the synovium, subchondral bone, ligaments, and muscles through intricate pathomechanisms. Whereas it was initially depicted as a predominantly aging-related and mechanically driven condition given its clear association with old age, high body mass index (BMI), and joint malalignment, more recent research identified and described a plethora of further factors contributing to knee OA pathogenesis. However, the pathogenic intricacies between the molecular pathways involved in OA prompted the study of certain drugs for more than one therapeutic target (amelioration of cartilage and bone changes, and synovial inflammation). Most clinical studies regarding knee OA focus mainly on improvement in pain and joint function and thus do not provide sufficient evidence on the possible disease-modifying properties of the tested drugs. Currently, there is an unmet need for further research regarding OA pathogenesis as well as the introduction and exhaustive testing of potential disease-modifying pharmacotherapies in order to structure an effective treatment plan for these patients.

## 1. Introduction

Osteoarthritis (OA) is a chronic musculoskeletal condition that primarily affects weight-bearing joints (such as the knees, hips, and spine) yet may involve the hands as well as other non-weight-bearing articular sites [1,2,3,4,5]. Genetic predisposition has been deemed relevant, however, more so in the hands and hips rather than in knee OA [1,2,3,4,5,6]. Moreover, certain racial and gender-related differences were also reported [6,7]. Nevertheless, OA remains a multifactorial whole-joint disease, the appearance and progression of which involves the alteration of articular cartilage as well as the synovium, subchondral bone, ligaments, and muscles through intricate pathogenic mechanisms [1,2,3].

Whereas it was initially depicted as a predominantly aging-related and mechanically driven condition given its clear association with old age, high body mass index (BMI), and joint malalignment, more recent research identified and described a plethora of further factors contributing to knee OA pathogenesis [6,7,8,9,10].

**Figure 1:**
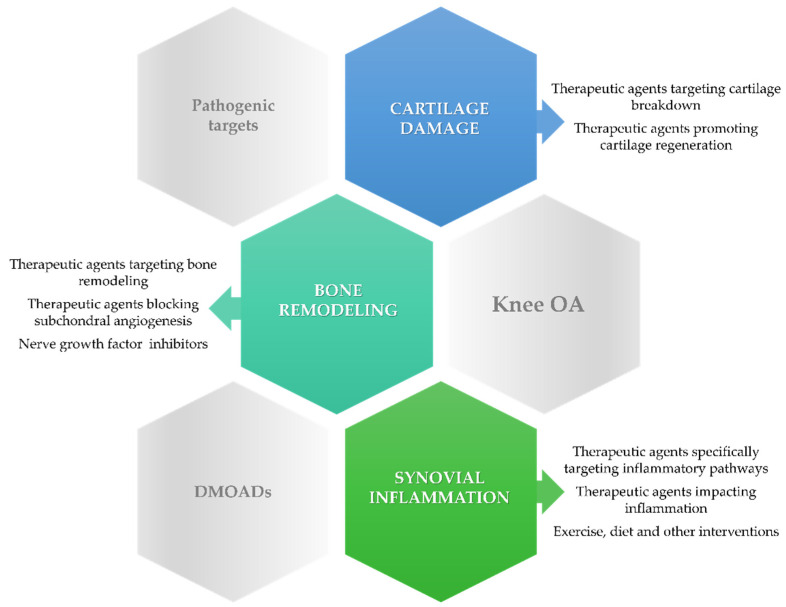
Therapeutic interventions targeting cartilage breakdown, bone remodeling, and inflammation in knee osteoarthritis (OA).

Expert opinion in OA proposes case stratification, describing four phenotypes of the disease largely based on pathogenesis: mechanical, metabolic, osteoporotic, and inflammatory. Nonetheless, patient stratification could lead to more precise identification of the potential therapeutic targets yet demands a comprehensive evaluation pretreatment [5]. Novel findings on the mechanisms underlying the development of knee OA prompted the search for potential disease-modifying OA drugs (DMOADs) able to counteract the molecular pathways involved in cartilage degradation, inflammation, and bone remodeling (Figure 1). However, most therapeutic agents with potential disease-modifying properties have not yet proven their efficacy in slowing the progression of knee OA in clinical trials [6,7,8].

The present review aims to discuss the potentially disease-modifying therapeutic options targeting cartilage destruction, subchondral bone remodeling, and synovial inflammation in knee OA according to recent findings.

## 2. Therapeutic Approach to Cartilage Damage in Knee Osteoarthritis

**Figure 2:**
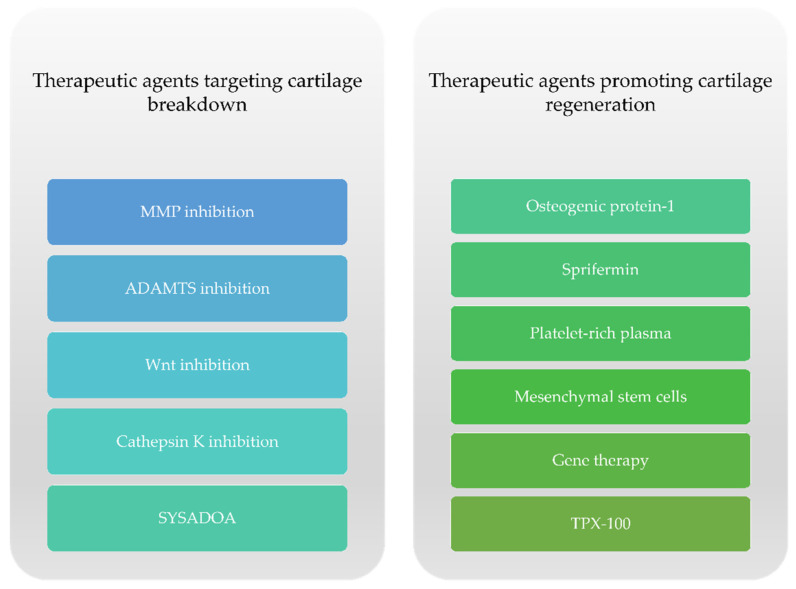
Therapeutic options targeting cartilage damage in knee OA.

The approval of DMOADs, according to regulatory guidelines from the United States Food and Drug Administration (FDA) and the European Medicines Agency (EMA) would need to meet the following conditions: slower loss in knee or hip joint space width (JSW) on x-ray and an appropriate symptomatic improvement [11]. Presently, there is no approved DMOAD despite the large amount of research conducted on the subject [12]. The currently available literature describes two subsets of potential DMOADs regarding cartilage damage in knee OA with respect to their specific actions: therapeutic agents targeting cartilage degeneration and drugs that support cartilage regeneration (Figure 2).

### 2.1. Matrix Metalloproteinase Inhibition

Matrix metalloproteinases (MMPs) and aggrecanases are known as the main proteinases responsible for matrix degradation in OA [13,14]. MMPs, a family of zinc-dependent enzymes, are recognized for their involvement in the degeneration of the extracellular matrix (ECM) [15]. They can be classified into the following groups: collagenases (MMP-1 and MMP-13), gelatinases (MMP-2 and MMP-9), stromelysins (MMP-3), metalloelastase (MMP-12), matrilysin (MMP-7), and membrane-type MMPs (MT-MMPs) [16].

In 2007, Krzeski et al. published a study of PG-116,800 (PG-530,742), an MMP-inhibitor, but no significant changes were observed in joint space width (JSW) of the knee or Western Ontario and McMaster Universities Osteoarthritis Index (WOMAC) scores at 1 year [17], yet important musculoskeletal side effects (named the “musculoskeletal syndrome” (MSS)) such as pain, loss of range of motion in large joints, joint swelling, stiffness, soft tissue pain, and Dupuytren’s contracture were observed in the treatment group. The exact cause of these reactions is not yet fully understood, but it is thought that chelation of zinc is involved in the process, seeing as the medication contains zinc-binding groups [18].

Given its notable pathogenic role in both OA as well as cartilage homeostasis, MMP-13 has been studied as a potential therapeutic target. ALS 1-0635 is a molecule that is free from zinc-chelating functional groups. Baragi et al. found a 67% deceleration in cartilage decline compared to the placebo in experimental animals in vivo [19].

Doxycycline is a broad-spectrum tetracycline antibiotic that also inhibits MMP activity, especially collagenase and gelatinase [20]. Nevertheless, a Cochrane review from 2012 stipulated that “the small benefit in terms of joint space narrowing is of questionable clinical relevance and outweighed by safety problems” [21].

### 2.2. ADAMTS Inhibition

In addition to MMPs, aggrecanases are also implicated in cartilage metabolism, acting as blockers of the aggrecan, the main proteoglycan of articular cartilage [22]. Aggrecan is a proteoglycan that incorporates chondroitin sulphate and keratan sulphate and is connected to a protein core. It links to another molecule, hyaluronan, thus creating stable large-molecular-weight molecules binding with a separate globular link [23]. In OA, the loss of aggrecan is an early event in the degradation of articular cartilage and results in the decrement of functional and structural ECM integrity followed by an irreversible loss of collagen [24].

Based on the fact that ADAMTS-4 (aggrecanase 1) and ADAMTS-5 (aggrecanase 2) are able to cleave proteoglycans (such as aggrecan—the main component of articular cartilage), both of these molecules have been studied as potentially disease-modifying targets in OA. A humanized anti-ADAMTS-5 antibody (GSK2394002) was assessed in a study conducted by Larkin et al. The authors demonstrated the efficacy of this drug in preventing constitutional destruction as well as in decreasing mechanical pain. Nevertheless, systemic administration resulted in significant side effects (increased mean arterial pressure and cardiac ischemia) and therefore was not approved as a DMOAD [25]. CRB007, a chimeric murine/human IgG4 anti-ADAMTS-5 monoclonal antibody, displayed disease-modifying properties in animal models of OA in the study published by Chiusaroli et al. However, further extensive research is needed [26].

Huang et al. described several small molecular aggrecanase inhibitors that demonstrated chondroprotective activity in patients with OA. One of these compounds is AGG-523, a per os ADAMTS-4 and -5 inhibitor [27]. Animal models of OA demonstrated a reduced level of aggrecan fragments in joints [28]. AGG-523 was part of two Phase I studies, but these trials were discontinued due to unknown reasons [27].

A derivative of 5-(1H-pyrazol-4-yl) methylene)-2-thioxothiazolidin-4-one has been suggested to exhibit important activity against ADAMTS-5. Together with a hyaluronic acid hydrogel (HAX), this molecule was injected into the knees of rats. The results of the study indicate that ADAMTS-5 could be a promising target for disease-modifying OA treatment [29]. A 2017 study reported the effects of per os GLPG1972/S201086, a potent inhibitor of ADAMTS-5. GLPG1972/S201086 confirmed the important protective effect on cartilage and subchondral bone in posttraumatic OA by remarkably diminishing cartilage proteoglycan loss, cartilage impairment, and subchondral bone sclerosis. Furthermore, the safety parameters evaluated indicated that the drug was well tolerated [30]. A Phase II trial assessing the efficacy and safety of 3 doses of per os GLPG1972/ S201086 once daily in patients with knee OA was completed in July 2020, yet the final results have yet to be reported [31].

### 2.3. Wnt Inhibition

Wang et al. described Wnt as a glycoprotein with extracellular position, for which signaling engages 19 Wnt genes and receptors that are capable of managing canonical β-catenin-dependent and noncanonical β-catenin-independent signaling pathways [32]. The abovementioned pathways are responsible for various biological aspects involving the cartilage, thus confirming their pathological role in OA [33,34].

Small molecules such as XAV-939 and SM04690 were discovered and studied as potential inhibitors of Wnt signaling in patients with knee OA. Certain Phase I, II, and IIb studies highlighted the positive effects of SM04690 in terms of pain reduction, functional impairment, and JSW in addition to a seemingly good safety profile. These data suggest that SM04690 could be considered for use as a disease-modifying agent in patients with moderate to severe symptomatic knee OA [35,36,37].

Takada et al. and Grossmann et al., in their respective studies of StAx-35R (a staple β-catenin-binding domain of Axin) and SAH-Bcl9 (a staple peptide derived from the Bcl9 homology domain-2), described the involvement and role in β-catenin transcriptional activity [38].

Another molecule involved in Wnt/β-catenin signaling is LRP5 (lipoprotein receptor-associated protein 5), which stimulates catabolic factors responsible for OA cartilage destruction and inhibits the anabolic factor type II collagen. A study on LRP5-knockdown mice confirmed the benefits on cartilage [39,40].

Lorecivivint inhibits the Wnt signaling pathway while also suppressing CLK2 (CDC-like kinase 2) and DYRK1A-mediated (dual-specificity tyrosine phosphorylation-regulated kinase 1A) phosphorylation of SIRT1 and FOXO1, both involved in Wnt/β-catenin activity. The drug has been shown to be relatively safe and well-tolerated, with important outcomes regarding cartilage destruction and inflammation, by decreasing catabolic enzymes and blocking the inflammatory process [41]. Studies on patients with knee OA have demonstrated the favorable effects of lorecivivint on reducing symptoms (notably pain), and improving physical function and patient global assessment [42].

### 2.4. Cathepsin K Inhibition

Cathepsin K is a lysosomal cysteine protease found in activated osteoclasts and chondrocytes (as well as other cell types) and has been shown to be involved in cartilage degradation (by destroying types I and II collagen and aggrecan found in the cartilage) and bone resorption [43,44].

Balicatib (AAE581), a cathepsin K inhibitor, was investigated in the treatment of patients with knee OA. The important side effects (skin rashes and dermal fibrosis) surpassed the beneficial outcome on both cartilage and bone, therefore leading to suspension of the study [45].

MIV-711 is described as being a selective and reversible agent that inhibits cathepsin K activity. MIV-711 holds an important role in decreasing serum CTX-I (a marker of bone turnover) and CTX-II levels (a marker of cartilage turnover), thus being implicated in both bone resorption and cartilage impairment [46]. MIV-711 was investigated in a multicenter, randomized, placebo-controlled, double-blind, three-arm parallel, Phase IIa study in patients with knee OA. The results showed that the reduction in medial femoral cartilage thickness was considerably reduced at 26 weeks. However, the medial tibia cartilage loss was not significantly changed. There was no substantial difference in pain reduction and quality of life scores or biomarker CTX-I and CTX-II values. The most important adverse events described were musculoskeletal symptoms, rashes, and infections. During the 6-month follow-up period, Conaghan et al. reported not finding significant benefits regarding OA-related symptoms. However, there is a need for further confirmation of MIV-711 as a DMOAD in OA through long-term trials [47].

### 2.5. Osteogenic Protein-1

BMP-7 (bone morphogenetic protein-7 or osteogenic protein-7), a member of the (TGF)-β superfamily, is a growth factor and is considered a possible therapeutic target in the process of restoration of damaged cartilage. Research analyzed BMP-7 (eptotermin-alpha) in the treatment of knee OA. A Phase I trial reported the amelioration of clinical symptoms and no dose-limiting toxicity. A Phase II trial and a Phase I trial also investigated BMP-7, but results have not yet been published [48].

### 2.6. Sprifermin

Sprifermin (AS902330) is a recombinant human fibroblast growth factor-18 (rhFGF18). According to published data, sprifermin has positive effects on cartilage by stimulating cell multiplication and ECM constituent production [49,50]. On intraarticular administration, it binds FGFR3 receptors from the cartilage [51,52].

The study of Dahlberg et al. (first in-human trial, randomized, double-blind, placebo-controlled study) investigated patients who proposed joint arthroplasty due to severe OA of the knee. However, no significant differences in terms of symptom improvement were described between the treatment group and the placebo cohort [53].

Preliminary results at 3 years of an important study of sprifermin were presented by Hochberg et al. in 2019. The FORWARD study, a 5-year Phase II, dose-ranging, randomized trial investigated the effect of intraarticular injections of sprifermin administered every 6 or 12 months versus the placebo. The consequence of drug injection was a lower mean cartilage thickness loss compared to natural evolution (total femorotibial joint as well as in the medial, lateral, central medial, and central-lateral regions). Importantly, the FORWARD study followed specifically structural progression, not other clinical issues associated with knee OA development and progression [54].

### 2.7. Platelet-Rich Plasma

Platelet-rich plasma (PRP) incorporates granules containing growth factors (transforming growth factor-β (TGF-b), platelet-derived growth factor (PDGF), insulin-like growth factor (IGF_, vascular endothelial growth factor (VEGF), and fibroblast growth factor (FGF)), cytokines, chemokines, and other mediators implicated in mesenchymal stem cell (MSC) proliferation and production of ECM and collagen, thus playing an important part in cartilage restoration [55,56].

Several randomized clinical trials have been completed in patients with knee OA, with intraarticular PRP being compared with hyaluronic acid. In terms of efficacy, the results of these studies revealed a favorable outcome in reducing symptoms and increasing mobility in these patients (with a mean duration of 12 months). Furthermore, no serious adverse effects were reported, suggesting a good safety profile for PRP [57,58,59]. However, there were a lot of discrepancies between these studies specifically in the protocols, methods, and patient characteristics; thus, it is challenging to compare all the data obtained [57].

### 2.8. Mesenchymal Stem Cells

Another promising treatment for cartilage repair seems to be MSCs derived from bone marrow, adipose tissue, or the umbilical cord. Studies have demonstrated that, compared with MSC injection, cell implantation has better results in patients with knee OA. Furthermore, there is a similar safety profile between autologous and allogenic MSCs [60,61].

A systematic review published by Jevotovsky et al. in 2018 evaluating 61 studies with MSCs as OA treatment concluded that MSC therapy has a favorable effect on OA patients but that there is limited high-quality evidence as well as a lack of long-term follow-up [62].

In 2015, Vega et al. presented a 1-year clinical trial that included patients with knee OA treated with intraarticular injections of allogeneic bone marrow MSCs. The results of this study showed considerable improvement in pain and functional indices, with a significant decrease in poor-quality cartilage areas and sustained augmentation of cartilage quality in the affected regions (quantified by magnetic resonance imaging (MRI) T2 mapping) [63].

An interesting aspect was represented by the possibility of treating MSCs before administration in order to boost the modulatory effect in OA. An example is combining MSCs with an inhibitor of signal transducer and activator of transcription 3. Under these conditions, the pro-inflammatory state was shown to be improved and the results of the treatment were more encouraging [64].

### 2.9. Gene Therapy

Gene therapy could be a promising alternative to OA treatment, mainly because of its long-term results on OA cartilage. Studies underline that intraarticular gene transfer treatment is a feasible option for patients suffering from OA compared to systemic administration with respect to safety, bioavailability, and direct targeting of the pathological site [65,66].

### 2.10. TPX-100

TPX-100, a peptide derived from matrix extracellular phosphoglycoprotein (MEPE), has expressed positive function when inducing articular cartilage regeneration in animal models. It is considered that this molecule facilitates cartilage formation only in areas with defects, without ectopic bone tissue formation [30].

A Phase II clinical study evaluating TPX-100 in patients with patellofemoral OA indicated significant amelioration and clinically efficient improvement in KOOS (Knee injury and Osteoarthritis Outcome Score) and WOMAC scores. Furthermore, promising effects were obtained concerning tibiofemoral cartilage thickness and volume at 6 and 12 months. Importantly, this performance was preserved for 30 months. The use of symptomatic treatment (nonsteroidal anti-inflammatory drugs (NSAIDs) and analgesics) diminished significantly across the study [67].

### 2.11. Symptomatic Slow-Acting Drugs for Osteoarthritis

A group of drugs known as SYSADOA (symptomatic slow-acting drugs for OA) have been used in patients with OA in order to improve clinical symptoms (pain and morning stiffness) as well as to slow progression of the disease. Among SYSADOA, chondroitin sulphate, glucosamine, and diacerein are the most widely studied. Using quantitative MRI (qMRI), clinical trials have demonstrated the contribution of chondroitin sulphate in decreasing cartilage volume loss [7,8]. Chondroitin sulphate influences proteoglycan metabolism and plays an important role in the balance of anabolic and catabolic processes at the ECM level. Chondroitin sulphate also decreases a series of proinflammatory factors with destructive properties in subchondral bone osteoblasts [9,10].

## 3. Therapeutic Approach to Bone Remodeling in Knee Osteoarthritis

**Figure 3:**
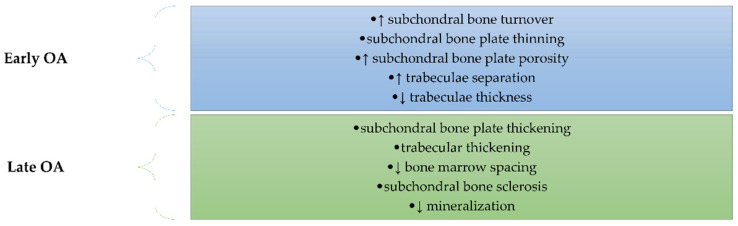
The main structural changes in the subchondral bone occurs in OA depending on the stage of the disease (early or late). In the early stage, there is an increase in subchondral bone turnover characterized by thinning of the subchondral bone plate and by increasing porosity associated with impairment of the trabeculae: thickness decreasing and separation increasing. The late stage of OA is characterized by thickening of the plate and trabecular layers, by decreasing bone marrow spacing, by sclerosis of the subchondral bone, and by decreased mineralization.

Subchondral bone remodeling plays a very important role in OA, mediating and preceding cartilage damage [68]. The structural changes in the subchondral bone are different depending on the stages of OA (Figure 3). Thus, in the early stage, there is an increase in subchondral bone turnover characterized by thinning of the subchondral bone plate and by increasing porosity associated with impairment of the trabeculae: thickness decreasing and separation increasing [69]. The late stage of OA is characterized by thickening of the plate and trabecular layers, by decreasing bone marrow spacing, and by sclerosis of the subchondral bone. Even if bone thickening occurs, there is insufficient bone mineralization due to a decreased calcium and collagen ratio followed by increased bone turnover [70,71,72,73,74,75].

Furthermore, the changes in subchondral bone are related to several signaling pathways such as the Wnt/β-Catenin, TGF-β/Smad, RANK/RANKL/OPG (Receptor activator of nuclear factor kappa-Β, RANK ligand, and osteoprotegerin), and MAPK (mitogen-activated protein kinase) signaling pathways [76,77,78,79,80,81,82,83,84]. Moreover, identification of the molecular pathways involved in OA-related bone remodeling has led to certain therapeutic agents targeting the mechanisms considered as possible DMOADs [85,86,87,88,89,90].

**Figure 4:**
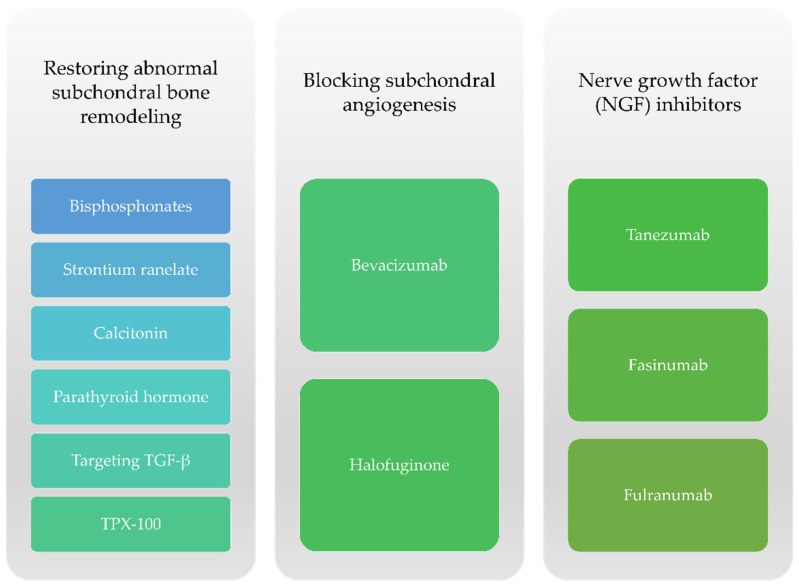
Therapeutic options targeting subchondral bone remodeling for knee OA.

Regarding subchondral bone changes, therapies aim to block bone resorption caused by osteoclasts as well as other mechanisms such as novel angiogenesis or particular neuronal factors responsible for the onset of pain (Figure 4).

### 3.1. Bisphosphonates

Bisphosphonates are antiresorptive drugs that can slow bone remodeling through the inhibition of osteoclasts. There are many published data that have shown pain relief, reduced bone destruction, and an improvement in joint structure following these treatments, but the clinical results are not very clear [91,92].

Alendronate, by inhibiting subchondral bone loss, caused an improvement in the structure of articular cartilage [93]. Other data showed an improvement of WOMAC pain score, an increase in bone mineral density, and a decrease in markers of bone destruction after alendronate administration for 2 years in patients with OA [91].

Risedronate use has not been shown to be clinically and radiologically effective in a group of 2483 patients with knee OA after 2 years of follow-up [94]. On the other hand, Spector et al. showed an improvement in pain and a decrease in subchondral remodeling following risedronate treatment [95].

The favorable effect of zoledronic acid was highlighted in a study that included 59 patients with knee OA. After 6 months of treatment, a significant reduction in pain was observed as well as a reduction in bone marrow lesions detected by magnetic resonance imaging [96]. Less favorable results (in relieving pain and loss of cartilage volume) after 24 months of treatment with zoledronic acid in knee OA cases were published by Aitken [91,92].

Clodronate, a non-amino bisphosphonate, appears to have a favorable effect in patients with knee OA, acting by increasing the secretion of SOX9, the transcription factor responsible for progenitor stem cell chondrogenic commitment. A 12-week, randomized, placebo-controlled study that included 80 cases of knee OA showed an improvement in pain and decreases in WOMAC score and in the need for analgesic medication after once weekly intraarticular injection of 2 mg clodronate [97].

### 3.2. Strontium Ranelate

Strontium ranelate can restore the abnormal subchondral bone remodeling by decreasing the activity of osteoclasts and by favoring the mineralization of new bone. After 3 years of treatment, patients with knee OA had an improvement in joint space narrowing compared to placebo [98]. At a dose of 1800 mg/day, there was a decrease in chondrocyte apoptosis and an improvement in cartilage matrix [99]. At a higher dose—2000 mg/day—strontium ranelate showed better effects in terms of loss of cartilage volume and regarding the improvement of bone marrow lesions [100].

### 3.3. Calcitonin

Calcitonin, a hormone secreted by parafollicular thyroid cells, binds to specific receptors on osteoclasts and inhibits their activity, thus reducing damage to the subchondral bone in OA [101,102]. In patients with knee OA, the use of oral calcitonin has been shown to have an effect in relieving pain and in increasing cartilage volume compared to placebo [103]. Due to the high risk of neoplasms, the use of calcitonin for short periods of time is recommended [104].

### 3.4. Cathepsin K Inhibition

Cathepsin K inhibitors are antiresorptive drugs that inhibit osteolytic protease of osteoclasts. There are several animal studies that have shown the beneficial role of these molecules in stopping the progression of OA [105,106]. In studies, there are 2 tested molecules: an oral inhibitor called MIV-711, for which structural changes in the joints were not considered, and balicatib (AAE581) used in the knee OA, in which narrowing of the articular space and the cartilage volume followed [107,108].

### 3.5. Parathyroid Hormone

Teriparatide, a recombinant human parathyroid hormone (PTH), is a bone anabolic therapy that regulates endochondral ossification and inhibits chondrocyte hypertrophy [109]. Systemic administration of teriparatide can inhibit cartilage degradation and abnormal chondrocyte differentiation, can stimulate regeneration of the matrix, and can improve the structure of the subchondral bone [110,111]. There are several ongoing studies that aim to highlight the beneficial effects of this molecule on both subchondral bone and joint cartilage, being assimilated as chondroregenerative therapy.

### 3.6. Trasforming Growth Factor β Inhibition

TGF-β gives positive feedback with the Wnt signaling pathway, favoring the differentiation of chondrocytes and osteoblasts [111]. In OA, TGF-β is secreted in excess by osteoblasts and causes the development of osteophytes [112]. Using a murine model, studies have highlighted the role of neutralizing TGF-β antibodies in stopping OA progression [113]. Other data have shown that implantation of an anti-TGF-β antibody (1D11) in alginic acid microbeads in the subchondral bone or deletion of the TGF-β type II receptor (TβRII) inhibits Smad2/3 phosphorylation in osteoblastic precursors, protecting the osteochondral unit [114].

### 3.7. TPX-100

MEPE is a bone protein secreted by osteocytes, with a negative role in regulating bone mineralization and involved in remodeling the subchondral bone [115]. A 23-aminoacid derived from MEPE (TPX-100) has been shown to be effective in cases of moderate femuro-patellar OA, leading to significant improvement in WOMAC scores. However, there was no evidence of structural improvement after 12 months of treatment [116].

### 3.8. Subchondral Angiogenesis Inhibition

Subchondral angiogenesis acts as a bridge between the articular cartilage and the subchondral bone. Blocking neoangiogenesis, the data showed a decrease in subchondral bone loss and a reduction in cartilage degradation [117]. Numerous factors such as VEGF (vascular endothelial growth factor), TGF-β1, PDGF-BB (platelet-derived growth factor BB monomer), and SLIT3 participate in the process of angiogenesis in the subchondral bone [117]. Halofuginone stops the action of TGF-β1 by inhibiting Smad2/3, thus blocking subchondral angiogenesis [118]. Bevacizumab, an antibody against VEGF, has been shown to be effective in reducing the formation of subchondral blood vessels, thereby inhibiting chondrocyte hypertrophy [119].

### 3.9. Nerve Growth Factor (NGF) Inhibition

Abnormal remodeling of the subchondral bone is associated with the presence of particular neural factors that cause innervation of sensory nerve structures in cases of OA [120]. NGFs are secreted by preosteoclasts, being triggers of subchondral bone innervation in OA [121]. NGF inhibitors such as tanezumab, fasinumab, or fulranumab have been shown to be effective in ameliorating joint pain and function in patients with OA [122,123]. For tanezumab, the data showed a significant improvement in pain [122,123], while fasinumab is still under investigation [124].

## 4. Therapeutic Approach to Synovial Inflammation in Knee Osteoarthritis

**Figure 5:**
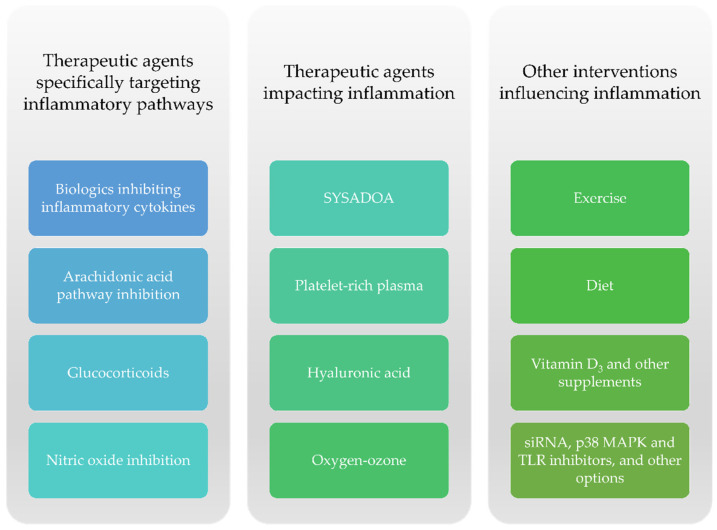
Drugs, dietary supplements, and other interventions contributing to a reduction in inflammation in knee OA.

The appearance of low-grade inflammation remains one of the most prominent features in OA pathogenesis. Proinflammatory cytokines such as IL-1, IL-6, and TNFα may be upregulated and may promote the release of ROS (reactive oxygen species), MMPs, and ADAMTS [125]. Though numerous emerging drugs were tested in degenerative joint conditions, a few studies focused specifically on their impact on knee OA-related inflammation (systemic or synovial fluid levels of inflammatory markers, the presence of joint effusion, or prolonged morning stiffness) [126,127,128,129,130]. Nevertheless, several drugs and dietary supplements as well as lifestyle interventions (diet and physical exercise) have been shown to exhibit disease-modifying properties in knee OA (Figure 5).

### 4.1. Biologics Targeting Proinflammatory Cytokines

Whereas they are more commonly used in immune-inflammatory diseases such as rheumatoid arthritis, psoriasis, ankylosing spondylitis, juvenile idiopathic arthritis, and inflammatory bowel diseases, certain TNFα inhibitors have also been studied in knee OA [131]. An open-label study including 56 patients with knee OA compared the efficacy of a single intraarticular injection with 10 mg adalimumab (ADA) versus 25 mg hyaluronic acid (HA), obtaining better results in terms of pain and functional impairment in the TNF inhibitor subgroup compared to their counterparts (Table 1) [132]. However, the follow-up period was short (4 weeks) and all patients were under concomitant treatment with systemic NSAIDs (celecoxib 200 mg daily) [132].

Another research conducted on 39 patients suggested that intraarticular injections with etanercept (ETN) could be superior to hyaluronic acid for pain relief in moderate to severe knee OA. While this was true for WOMAC pain score improvement at 4 weeks, the differences in mean VAS (Visual Analogue Scale) levels between the two study groups failed to reach statistical significance at the end of the follow-up period [133].

A multicenter, randomized, double-blind, placebo-controlled study of 50 mg and 150 mg intraarticular anakinra (ANR) versus placebo found no significant benefits for ANR over 12 weeks [134]. Wang et al. conducted a placebo-controlled, randomized, double-blind, ascending dose research focused on anti-interleukin-1α/β dual variable domain immunoglobulin ABT-981 in patients with symptomatic knee OA, identifying lower levels of high-sensitivity C-reactive protein (hsCRP) as well as IL-1α and IL-1β in the treatment group [135].

### 4.2. Arachidonic Acid Pathway Inhibition

The arachidonic acid pathway employs cyclooxygenase enzymes (COX) and prompts the release of proinflammatory prostaglandins. The latter are also involved in pain mediation, which is why NSAIDs are widely prescribed as symptomatic treatment in OA [136]. However, certain NSAIDs have additionally been considered for use as disease-modifying agents [137,138]. A study performed on knee OA patients examined the impact of celecoxib, ibuprofen, and diclofenac on synovial fluid proinflammatory cytokine (TNFα, IL-6, and IL-8) and VEGF expression [139]. Aside from significant pain relief, NSAID therapy (particularly higher doses) showed notable beneficial effects in reducing inflammatory markers in patients’ synovial fluid, thus suggesting that these drugs could be regarded as having disease-modifying properties [139]. Nevertheless, the numerous adverse events associated with prolonged administration of systemic NSAIDs limit their use primarily in older individuals and persons with comorbid conditions [140].

The dual hindrance of COX2 and 5-lipooxygenase (LOX) diminished the release of prostaglandin E2, leukotriene B4, collagenase-1, cathepsin, MMP-13, and IL-1β [141]. Moreover, COX/LOX blockade diminished OA-related cartilage degradation and synovial hypertrophy in preclinical studies [138,141]. Licofelone is a COX/LOX inhibitor that displayed a protective effect on knee cartilage volume loss in a multicenter clinical trial [142].

### 4.3. Glucocorticoids

Glucocorticoids exhibit potent anti-inflammatory effects and have been shown to reduce pain and pain-related functional impairment in patients with OA. However, intraarticular or systemic corticosteroid administration has seldom been investigated with respect to its effect on clinical or paraclinical indicators of inflammation in knee OA (joint effusion and stiffness, synovial fluid inflammatory markers, and imaging tests) [143,144,145]. Furthermore, the risk–benefit ratio for using intraarticular glucocorticoids in knee OA remains a matter of discussion among researchers, clinicians, and medical organizations alike [146].

Nevertheless, a study evaluating a combination of triamcinolone acetonide and sodium hyaluronate was superior to intraarticular saline in improving joint stiffness, pain, and function in patients with knee OA with no or minimal to moderate joint space narrowing (Kellgren–Lawrence grades 1–3) [147]. A recent study investigating the effect of intraarticular injections with triamcinolone found that the latter reduced joint effusion in patients with knee OA [148].

### 4.4. Symptomatic Slow-Acting Drugs for Osteoarthritis

The DISSCO study examined the efficacy of diacerein (diacetylrhein, an anthraquinone derivative, and SYSADOA) compared to celecoxib on knee osteoarthritis. The multicenter randomized trial concluded that diacerein was non-inferior to celecoxib. The percentage of patients experiencing joint effusion diminished by half over time, however, without notable discrepancies between the two treatment groups [149]. In rat synovial cells, diacerein reduced the mRNA levels of inflammatory cytokines (TNFα, IL-1, and IL-6), COX2, cartilage-degrading enzymes (MMP-3 and MMP-13), and ADAMTS-5 in a dose-dependent manner while also raising those of certain anti-inflammatory factors (IL-4 and IL-10). Moreover, the analysis of biopsy samples taken from rats with monosodium iodoacetate-induced OA as well as imaging tests (computed tomography) showed that the administration of diacerein-loaded nanoparticles may protect against joint destruction [150].

Other SYSADOAs such as glucosamine and chondroitin sulphate have also been thought to display anti-inflammatory properties together with benefits regarding cartilage degradation in patients with knee OA, yet results remain discrepant across studies [7,8,9,10].

### 4.5. Nitric Oxide Inhibition

Nitric oxide (NO) together with inducible NO synthase (iNOS, an enzyme that participates in NO synthesis) are involved in OA-related inflammation, cartilage damage, and cellular injury. The release of proinflammatory cytokines such as TNFα, IL-1β, IL-17, and interferon γ (IFNγ) has been shown to upregulate the expression of iNOS, thus emphasizing the link between inflammation and oxidative stress [151,152].

Scientific evidence highlighting the involvement of iNOS in OA pathogenesis prompted testing of iNOS inhibitors in human subjects. A randomized double-blind placebo-controlled trial of the iNOS inhibitor cindunistat (50 or 200 mg daily) was conducted on patients with symptomatic knee OA. In the Kellgren–Lawrence grade 2 subgroup, cindunistat 50 mg daily was associated with a delay in joint space narrowing at 48 weeks compared to the placebo. Nevertheless, these results were not maintained at 96 weeks [153]. Furthermore, the Kellgren-Lawrence grade 3 cohort did not show a significant improvement with respect to slowing knee OA progression with iNOS inhibition, irrespective of cindunistat dose [153].

### 4.6. Platelet-Rich Plasma

PRP is an autologous formulation containing concentrated blood-derived platelets and growth factors that promotes synoviocyte differentiation and proliferation, with studies reporting significant benefits in OA of the knee [154,155]. Moreover, PRP is thought to be rich in bioactive molecules involved in tissue repair and regeneration and to wield anti-inflammatory properties by lowering synovial fluid proinflammatory marker levels [156,157,158,159]. Notably, some authors support the use of leukocyte-poor PRP (LP-PRP), arguing that the presence of white blood cells in intraarticular formulation may generate pro-inflammatory effects [160]. PRP has been deemed safe for use and has demonstrated promising results in different chronic rheumatic conditions and injury-related joint damage [161]. In knee OA, PRP displayed positive effects in improving pain, joint function, and quality of life according to recently published meta-analyses [157,162].

In young adults (18–30 years of age) diagnosed with knee OA, intraarticular PRP therapy led to a statistically significant decrease in IL-1β, IL-6, TNFα, IL-17A, RANKL, and IFNγ levels (Table 2) [163]. A study performed on eutrophic individuals (BMI between 22–25 kg/m^2^) aged between 42 and 79 years comparing intraarticular PRP, HA, and the combination between PRP and HA found that IL-1β, TNFα, TIMP1, and MMP-3 values were diminished in the PRP group at 6 months posttreatment [164]. Nevertheless, the association of PRP and HA held the best results in reducing proinflammatory markers [164].

### 4.7. Hyaluronic Acid

A randomized, double-blind, placebo-controlled study of obese patients with knee OA examined the outcomes of a per os preparation containing HA (70%) and other glycosaminoglycans (GAGs) [165]. At 3 months, the treatment group exhibited a significant decrement in TNFα, IL-1α and IL-1β, IL-6, IL-17α, IFN, and GM-CSF (granulocyte-macrophage colony-stimulating factor) levels, while the placebo cohort demonstrated notably higher synovial fluid concentrations of proinflammatory cytokines as well as leptin [165].

A recent research comparing the efficacy of intraarticular HA to ozone (O_3_) found that both reduced joint stiffness in patients with knee OA [166]. Other published studies aimed to investigate the possible differences between intraarticular HA and biologics, PRP, or O_3_ (Table 3) [132,133,164,165,166]. Xu et al. reported positive results for a PRP + HA combination with respect to the reduction in proinflammatory cytokine values and MMP-3 [164].

### 4.8. Oxygen–Ozone

A study of intraarticular injections of oxygen–ozone (O_2_–O_3_) versus triamcinolone in knee OA determined that joint effusion was notably reduced in both treatment arms [148]. Whereas the decrement in WOMAC and VAS values were in favor of the O_2_–O_3_ group, the differences compared to corticosteroid-treated patients did not reach statistical significance with respect to the reduction in joint effusion on ultrasound [148]. Other published research reported finding a reduction in joint stiffness in knee OA patients following treatment with O_3_ (Table 4) [148,166,167].

### 4.9. Exercise, Diet, and Supplements

Diet and lifestyle changes have also been shown to reduce inflammation in knee OA. The IDEA (Intensive Diet and Exercise for Arthritis) study enrolled over 400 overweight and obese individuals (BMI between 27–40.5 kg/m^2^) with symptomatic knee OA. The research subjects were randomized to three intervention groups (exercise, diet, or diet and exercise) and followed the program for 18 months [168]. Both diet as well as the combination of diet and exercise significantly lowered serum inflammatory marker levels [168].

Another research performed on women with knee OA examining the role of resistance training and 1000 mg daily nanocurcumin (turmeric) showed that the latter reduced synovial fluid NO concentrations by over 26% and collagenase II by circa 4.7% [169]. However, the authors commented that the short duration of the study (16 weeks) could explain the lack of statistically significant differences between the 3 intervention groups (resistance exercise, curcumin supplementation, and exercise and supplementation) [169].

Vitamin D-deficient individuals were found to be at risk of developing knee OA [160]. The impact of vitamin D supplementation on pain relief, the improvement in functional parameters, and cartilage loss remain matters of debate [170,171]. However, an MRI study analyzing the effect of 50,000 IU monthly vitamin D_3_ supplementation in patients with knee OA found that the placebo group exhibited a significant increase in effusion-synovitis volume over two years compared to the treatment cohort [172].

It has been stated that old age may be associated with intestinal dysbiosis and increased gut permeability, which could promote a “leakage” of gut microbes into tissues, thus leading to a chronic proinflammatory state [173,174]. Tsai et al. aimed to characterize the immunological signature of the microbiome in osteoarthritic knee synovium, finding marked discrepancy in microbial abundance in patients compared to healthy controls [175]. Whereas intestinal dysbiosis has been linked to synovial inflammation in knee OA, the impact of probiotic use on disease progression have not been studied extensively in clinical trials [176,177]. Certain experimental studies reported promising results with respect to the administration of *Lactobacillus acidophilus* in rats. In this regard, *Lactobacillus acidophilus* prompted the diminishment in proinflammatory marker expression together with an increase in anti-inflammatory cytokines in monosodium iodoacetate-induced OA. Moreover, *Lactobacillus acidophilus* exhibited anti-nociceptive effects in experimental animals [178].

It has been stated that dietary polyphenols and herbal medicine may also exhibit anti-inflammatory properties and reduce iNOS expression in OA [151,179]. Scoparone is a compound extracted from *Artemisia capillaris* that may exhibit potent anti-inflammatory effects. A recent study proved that scoparone could repress IL-1β-induced activation of the PI3K/Akt/NF-κB pathway as well as COX2 and iNOS [180]. In murine models of OA, acteoside (withdrawn from *Ligustrum purpurascens* kudingcha tea) reduced the expression of TNFα, IL-6, and IFNγ and hindered the IL-1β-related activation of the JAK/STAT (janus kinase/signal transducer and activator of transcription) pathway [181].

Avocado soybean unsaponifiables were studied in knee OA, showing positive results. Moreover, avocado soybean unsaponifiables diminished the expression of iNOS, MMP-13, and TNFα in rat monosodium iodoacetate-induced OA [182].

### 4.10. Other Therapeutic Options Exhibiting Antiinflammatory Properties

The inhibition of TNFα and pan-cytokine small interference RNA (siRNA) led to a decrease in proinflammatory cytokines (IL-1, IL-6, IL-8, and TNFα), with promising findings being recorded in collagenase-induced murine models of OA [183,184,185].

P38 mitogen-activated protein kinase (MAPK), toll-like receptors (TLR2 and TLR4), and leptin have been considered potential targets for the development of new DMOAD options based on their experimentally shown involvement in OA-related inflammation [138]. Though in vitro and animal studies reported certain encouraging results, the efficacy of siRNA, p38 MAPK, TLR, and inflammatory adipokine blockade remains in need of further testing in clinical trials of knee OA [138].

Carboxymethyl chitosan (a chitosan soluble derivative) displays a significant physicochemical likeness to cartilage proteoglycans. In rat chondrocytes, increasing doses of carboxymethyl chitosan demonstrated iNOS inhibition and upregulated anti-inflammatory cytokine expression (IL-10) [186].

## 5. Conclusions

OA is a chronic and pathogenically multifaceted disease that remains a major cause of disability worldwide. Cartilage degradation, inflammation, and bone remodeling are presently regarded as therapeutic targets for the development of DMOADs. However, the pathogenic intricacies between the molecular pathways involved in OA prompted the study of certain drugs for more than one therapeutic target (amelioration of cartilage and bone changes, and inflammation). Most clinical studies regarding knee OA focus mainly on improvement in pain or joint function and thus do not provide sufficient evidence on the possible disease-modifying properties of the tested drugs. Currently, there is an unmet need for further research regarding OA pathogenesis as well as the introduction and exhaustive testing of potential disease-modifying pharmacotherapies in order to structure an effective treatment plan for these patients.

## Figures and Tables

**Table 1:** Proinflammatory cytokine blockers adalimumab, etanercept, anakinra, and ABT-981 in knee OA.

Reference	Compound	Intervention	Patients	Duration	Results
Wang [132]	Adalimumab (ETA)	10 mg intraarticular ADA + Celecoxib 200 mg/day versus 25 mg HA + Celecoxib 200 mg/day	ADA + Celecoxib (N = 28) HA + Celecoxib (N = 28)	4 weeks	The authors found a significant improvement in pain and functionality in the ADA group.
Ohtori et al. [133]	Etanercept (ETN)	10 mg intraarticular ETN versus 25 mg HA	ETN (N = 19) HA (N = 20)	4 weeks	An initial significant amelioration was obtained in the ETN group compared to HA (weeks 1 and 2), yet the results were not maintained at week 4.
Chevalier et al. [134]	Anakinra (ANR)	150 mg intraarticular ANR 50 mg intraarticular ANR versus Placebo	ANR 150 mg (N = 67) ANR 50 mg (N = 34) Placebo (N = 69)	12 weeks	A significant pain improvement was observed in the 150 mg ANR group compared to 50 mg ANR at day 4. Overall, intraarticular ANR did not demonstrate notable benefits, irrespective of the dose.
Wang et al. [135]	ABT-981	ABT-981 (various doses) versus Placebo	ABT-981 0.3 mg/kg fortnightly (N = 7) 1 mg/kg fortnightly (N = 7) 3 mg/kg fortnightly (N = 7) 3 mg/kg every 4 weeks (N = 7) Placebo (N = 8)	113 days (cohorts 1, 2, and 3) 127 days (cohort 4)	Mean hsCRP decreased through week 2 irrespective of ABT-981 dose/administration interval. While IL-1α and IL-1β were lower in the treatment group, serum vascular endothelial growth factor and MMP-9 did not demonstrate significant changes.

**Table 2:** Platelet-rich plasma (PRP) treatment in knee OA: anti-inflammatory properties.

Reference	Intervention	Patients	Duration	Results
Huang et al. [163]	2–14 mL intraarticular PRP weekly versus Placebo (10 mL saline solution) weekly	PRP (N = 310) Placebo (N = 56)	8 weeks	Significant improvements in plasma IL-1β, IL-6, TNFα, IL-17A, RANKL, and IFNγ were observed in the treatment group.
Xu et al. [164]	4 mL intraarticular PRP, 3 injections per knee, half-month interval 2 mL intraarticular HA, 3 injections per knee, half-month interval 4 mL PRP + 2 mL HA, 3 injections per knee, half-month interval	PRP (N = 40 knees) HA (N = 34 knees) PRP + HA (48 knees) N = 78 patients total (122 knees) N = 44 patients received bilateral injections	24 months	IL-1β, TNFα, TIMP1, and MMP-3 demonstrated significant decreases in the PRP group at 6 months posttreatment. Nevertheless, the PRP + HA group showed better results in this respect. Additionally, the PRP + HA cohort displayed IL-1β, TNFα, TIMP1, and MMP-3 inhibition at 12 months post-injection.

**Table 3:** Hyaluronic acid (HA) treatment in knee OA: anti-inflammatory properties.

Reference	Intervention	Patients	Duration	Results
Nelson et al. [165]	80 mg oral preparation of HA (70%) + other GAGs versus Placebo	Oral preparation of HA + other GAGs (N = 21) Placebo (N = 19)	12 weeks	The HA-treated cohort demonstrated a notable decrease in TNFα, IL-1α, IL-1β, IL-6, IL-17α, IFN, and GM-CSF values. Furthermore, the placebo group exhibited significantly higher synovial fluid concentrations of inflammatory cytokines as well as leptin (a proinflammatory adipokine).
Wang [132]	25 mg HA + Celecoxib 200 mg/day versus 10 mg intraarticular ADA + Celecoxib 200 mg/day	HA + Celecoxib (N = 28) ADA + Celecoxib (N = 28)	4 weeks	Changes in joint stiffness did not exhibit statistically significant differences between HA + Celecoxib and ADA + Celecoxib.
Ohtori et al. [133]	25 mg HA versus 10 mg intraarticular ETN	HA (N = 20) ETN (N = 19)	4 weeks	The HA group displayed significantly weaker results in terms of joint stiffness improvement compared to ETN during the follow-up period.
Xu et al. [164]	2 mL intraarticular HA, 3 injections per knee, half-month interval 4 mL intraarticular PRP, 3 injections per knee, half-month interval 2 mL HA + 4 mL PRP, 3 injections per knee, half-month interval	HA (N = 34 knees) PRP (N = 40 knees) HA + PRP (48 knees)	24 months	The cohort that received the HA + PRP combination demonstrated IL-1β, TNFα, TIMP1, and MMP-3 inhibition at one-year posttreatment.
Raeissadat et al. [166]	3 weekly intraarticular injections of 20 mg/2 mL HA versus 3 weekly intraarticular injections of 30 μg/mL O_3_ (10 mL)	HA (N = 74) O_3_ (N = 67)	24 weeks	There was a significant reduction in joint stiffness in the group treated with HA. However, these results were not significantly different from the O_3_-treated cohort.

**Table 4:** O_3_ and O_2_–O_3_ treatment in knee OA: anti-inflammatory properties.

Reference	Intervention	Patients	Duration	Results
Babaei-Ghazani et al. [148]	15 μg/mL intraarticular O_2_–O_3_ (10 mL) versus 40 mg intraarticular triamcinolone (1 mL)	O_2_–O_3_ (N = 31) Triamcinolone (N = 31)	12 weeks	The authors found an important reduction in joint effusion on ultrasound in both treatment arms at 3 months post-injection.
Lopes de Jesus et al. [167]	20 μg/mL intraarticular O_3_ (10 mL) versus Placebo (10 mL air)	O_3_ (N = 61) Placebo (N = 35)	8 weeks	There was a significant improvement in joint stiffness at 8 weeks in the treatment arm.
Raeissadat et al. [166]	3 weekly injections of 30 μg/mL intraarticular O_3_ (10 mL) versus 3 weekly injections of 20 mg/2 mL HA	O_3_ (N = 67) HA (N = 74)	24 weeks	Both groups demonstrated a notable amelioration in joint stiffness yet without significant discrepancies between the two treatment arms.

## Data Availability

Not applicable.
